# Resolution of Papilledema Following Ventriculoperitoneal Shunt or Endoscopic Third Ventriculostomy for Obstructive Hydrocephalus: A Pilot Study

**DOI:** 10.3390/medicina58020281

**Published:** 2022-02-13

**Authors:** Sukriti Das, Nicola Montemurro, Musannah Ashfaq, Dipankar Ghosh, Asit Chandra Sarker, Akhlaque Hossain Khan, Sharbari Dey, Bipin Chaurasia

**Affiliations:** 1 Department of Neurosurgery, Bangabandhu Sheikh Mujib Medical University, Dhaka 1205, Bangladesh; sukriti66@yahoo.com (S.D.); fahimshahriyer.1@gmail.com (A.H.K.); sharbaride8@gmail.com (S.D.); 2 Department of Neurosurgery, Azienda Ospedaliera Universitaria Pisana (AOUP), University of Pisa, 56100 Pisa, Italy; 3 Department of Neurosurgery, Bangladesh Medical College and University, Dhaka 1205, Bangladesh; musannah45@gmail.com (M.A.); dipankar.polas@gmail.com (D.G.); asitchandra.sarker@yahoo.com (A.C.S.); troexa@gmail.com (B.C.)

**Keywords:** endoscopic third ventriculostomy, ETV, neurosurgery, obstructive hydrocephalus, papilledema, ventriculoperitoneal shunt, VPS

## Abstract

*Background and Objectives:* Ventriculoperitoneal Shunt (VPS) and Endoscopic Third Ventriculostomy (ETV) are both gold standard procedures to reduce intracranial pressure (ICP) in patients with obstructive hydrocephalus, which often results in papilledema. This comparative study was carried out at the Department of Neurosurgery of Dhaka Medical College and Hospital to compare the efficacy of VPS and ETV in the resolution of papilledema in 18 patients with obstructive hydrocephalus. *Materials and Methods:* The success of CSF diversion was evaluated by a decrease in retinal nerve fiber layer (RNFL) thickness by optical coherence tomography (OCT) and modified Frisen grading of papilledema at the same time. The statistical analyses were carried out by using paired sample t test and the Spearman’s correlation coefficient test. The level of significance (*p* value) was set at <0.05. *Results:* After 7 days, both VPS and ETV were able to reduce RNFL thickness of both eyes with a *p* value = 0.016 (right eye) and 0.003 (left eye) in group A (VPS) and with a *p* value <0.001 (both eyes) in group B (ETV). Change of Frisen grading after CSF diversion was not satisfying for both the procedures with *p* value > 0.05. Further, the inter-group comparison between VPS and ETV showed no difference in decreasing RNFL thickness and modified Frisen grading (*p* value = 0.56). *Conclusion:* VPS and ETV procedures both appear very efficient in treating obstructive hydrocephalus, which in turn reduces papilledema in these patients. This paper is preliminary and requires further work.

## 1. Introduction

Obstructive hydrocephalus is the clinical condition characterized by enlargement of cerebral ventricles and associated symptoms caused by raised intracranial pressure (ICP). The main goal of treatment is to decrease raised ICP, either by ventriculoperitoneal shunt (VPS) or endoscopic third ventriculostomy (ETV) [1,2,3,4]. Endoscopic third ventriculostomy (ETV) and ventriculoperitoneal shunt (VPS) are two surgical methods used for the treatment of obstructive hydrocephalus to create an alternative pathway for CSF to flow out of the ventricles and around the brain (ETV) or in the peritoneum (VPS). Increased ICP in patients with obstructive hydrocephalus can cause various systemic symptoms, as well as neuro-ophthalmic complications. The most common neuro-ophthalmic complications are papilledema and secondary optic atrophy [5,6]. Papilledema is defined as an optic disc swelling secondary to raised intracranial pressure which usually presents bilaterally and may develop within hours to weeks. Papilledema from various causes of IH may develop at any age, in either sex, and in any racial or ethnic group [7]. Several larger neurosurgical series found papilledema in up to 60–80% of patients with cerebral tumors [8]. Classical symptoms include headache, usually worse when awakening, vomiting and blurring of vision. Possible conditions causing high intracranial pressure and papilledema include intracerebral mass lesions, cerebral hemorrhage, head trauma, meningitis, hydrocephalus, spinal cord lesions, impairment of cerebral sinus drainage, anomalies of the cranium, and idiopathic intracranial hypertension (IIH) [7]. Fundus examination shows hyperemia and swelling of the optic disc, blurring of the disc margin, blurring of the peripapillary retinal fiber layer and absence of spontaneous venous pulsations [9]. Brain imaging and even serial brain imaging may not be enough to decide whether the brain is under pressure. In such patients, identifying papilledema and reducing ICP are crucial in the clinical management of patients with obstructive hydrocephalus [10]. There are numerous tests used for the screening of papilledema, and the sensitivity and specificity of each of these objective tests is still being debated. Early diagnosis of papilledema can help in early intervention, thus preventing visual loss, and even death [11,12]. Papilledema can be graded clinically using a modification of Frisen criteria [13]. Funnell et al. [14] reported that patients with papilledema had higher ICP values than patients who did not develop papilledema (10.4 vs. 6.3 mmHg, respectively) and observed that Frisen grade was statistically correlated with ICP values, whereas patients with higher ICP values were more likely to develop papilledema. Optical coherence tomography (OCT) is a non-invasive imaging technique which obtains retinal images closely resembling histological preparations, and is useful in evaluating the peripapillary nerve fiber layer thickness. OCT can detect and quantify diffuse thickening of the retinal nerve fiber layer (RNFL) in eyes with papilledema [15]. Mean RNFL thickness (360° measurement) ranges from 46 to 106 µm in normal eyes [16]. Resolution of the papilledema is commonly thought to be a good clinical indicator of decreased ICP after surgery [17,18]. Although different studies reported different cost-effectiveness, availability of the procedures, complications, revision and both clinical and radiological outcomes between VPS and ETV [19,20,21], at present there is no clear consensus on which procedure is better. For this reason, we performed a comparative prospective study to investigate and compare the effects of VPS and ETV on the resolution of papilledema from raised ICP due to obstructive hydrocephalus and if any differences in RNFL thickness by OCT occurred between two groups. We measured the rate of resolution RNFL thickness by OCT in both VPS and ETV, and reported the regression of papilledema and the effectiveness of the procedures. To the best of our knowledge, few studies in the literature reported an objective estimate of the papilledema due to obstructive hydrocephalus, using an objective measurement of the thickness of the RNFL measured byoptical coherence tomography.

## 2. Materials and Methods

This prospective comparative study was carried out in the Department of Neurosurgery of Dhaka Medical College and Hospital, Dhaka, Bangladesh (protocol code: ERC-DMC/ECC/2019/390) within the period of June 2018 and June 2020. All 18 patients signed informed consent for this study. Inclusion criteria were patients older than 18 years old having papilledema due to obstructive hydrocephalus from any intracranial lesion, of any age and sex, admitted in hospital. The exclusion criteria were patients’ inability to cooperate with the ophthalmic examination, patients having previous surgery for obstructive hydrocephalus and patients having any other ophthalmic diseases. According to the selected criteria, the study recruited 18 patients (36 eyes) who had papilledema due to raised intracranial pressure from obstructive hydrocephalus. All 18 patients underwent surgery, either VPS or ETV, for obstructive hydrocephalus and were randomly and prospectively divided into due groups: group A (patients underwent VPS) and group B (patients underwent ETV). Retinal nerve fiber layer (RNFL) thickness measurement and modified Frisen grading (Table 1) with color fundal photography were obtained in all patients 3 to 4 h before VPS or ETV surgeries and 7 days postoperatively, then patients with tumors underwent surgery for tumor removal.

The success of the procedures (resolution or subsidence of papilledema = improvement of visual blurring) was assessed via modified Frisen grading by fundoscopy and via RNFL thickness measurement by optical coherence tomography (OCT). OCT is a noninvasive, high-resolution optical imaging technology based on interference between signal from an object under investigation and a local reference signal [22]. These measures were evaluated by a single ophthalmologist. Here, average decrease in RNFL thicknesses and mean grade change for Frisen grading were used for comparison. Improvements of clinical symptoms regarding ICP and decrease in RNFL thickness on OCT resulted in a good surgical outcome. This study was conducted using a super-luminescent diode scan with a center wavelength of 840 nm and an acquisition rate of 27,000 A-scans per second at an axial resolution of 5 μm to measure RNFL thickness. Post-operative CT scans were obtained in all cases to rule out any hemorrhagic complications.

## 3. Results

Nine patients underwent VPS (Group A) and 9 patients underwent ETV (Group B). The mean ages of patients in Groups A and B were 30.7 ± 10.5 years old (range 18–47 years old) and 33.1 ± 11.2 years old (range 19–50 years old), respectively. Primary pathologies in both groups were 4 cerebellopontine angle (CPA) tumors (2 in group A, 2 in group B), 4 posterior fossa tumors (3 in group A, 1 in group B), 6 aqueductal stenosis (2 in group A, 4 in group B), and 4 pineal tumors (2 in group A, 2 in group B). All the 18 (100%) patients had bilateral papilledema, and headaches, with vomiting in 11 (61.1%) cases, hearing loss in 6 (33.3%), and gait disturbances in 6 (33.3%) cases. Table 2 shows all patient details.

All 18 patient were evaluated by an ophthalmologist before VPS or ETV surgeries and 7 days postoperatively, then 12 out 18 patients (66.7%) underwent surgery for tumor removal. For this reason, follow-up were performed after 7 days. In group A (VPS), mean RNFL thickness was 159.8 ± 59.4 µm preoperatively and 128.4 ± 36.3 µm postoperatively for right eyes, whereas mean RNFL thickness was 189.00 ± 56.6 µm preoperatively and 124.2 ± 27.6 µm postoperatively for left eyes. The mean RNFL thickness in group B (ETV) for right eyes was 158.4 ± 30.7 µm preoperatively and 110.5 ± 27.5 µm postoperatively. For the left eyes, the mean RNFL thickness was 161.1 ± 45.2 µm preoperatively and 99.6 ± 21.6 µm postoperatively. Table 3 shows more details. Decrease in mean RNFL thickness after either VPS or ETV (7 days postoperatively) was statistically significant for both eyes in both groups (VPS and ETV). Table 4 shows more details. No differences were seen on the bilateral nature of the papilledema in both groups based on the tumor location. RNFL thickness usually varies with age, however it seems that obstructive hydrocephalus did not affect RNFL thickness according with age.

**Figure 1:**
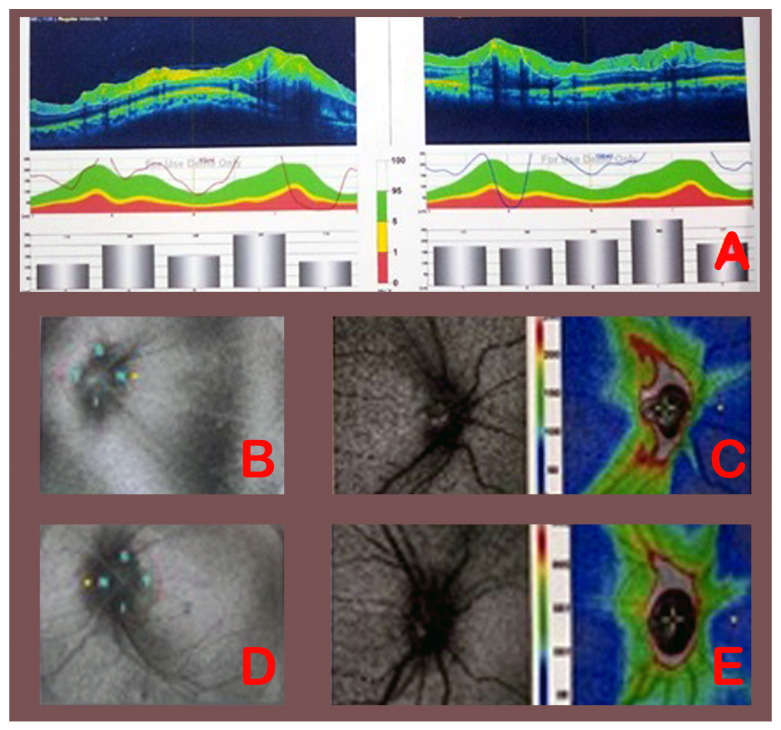
Pre-operative (**A**) OCT (RNFL, right eye 121 µm, left eye 239 µm). Pre-operative right (**B**) and left (**D**) eyes fundus and post-operative right (**C**) and left (**E**) eyes fundus and OCT (RNFL, right eye 109 µm, left eye 97 µm) after VPS in patient no. 3.

In group A (VPS), pre-operative modified Frisen grading of papilledema right eyes were grade 1 in 3 patients, grade 2 in 1 patient, grade 3 in 4 patients, and grade 5 in 1 patient (Table 5). Postoperatively, patients improved regarding Frisen grading: 6 patients were grade 1, 2 patients were grade 2, and only 1 patient had grade 3 papilledema, who started at grade 5. In the same way, pre-operative modified Frisen grading of papilledema in left eyes were grade 1 in 2 patients, grade 2 in 1 patient, grade 3 in 3 patients, grade 4 in 2 patients, and grade 5 in 1 patient. Patients also improved regarding Frisen grading after VPS: 7 patients were grade 1, 1 patient was grade 2, and 1 patient was grade 3. These changes to the severity of papilledema were statistically significant (*p* value = 0.028) for right eyes and not statistically significant for left eyes (*p* value = 0.333). Figure 1 shows pre-operative eyes and post-operative eyes fundus and OCT in patient no. 3, who was treated with VPS.

**Figure 2:**
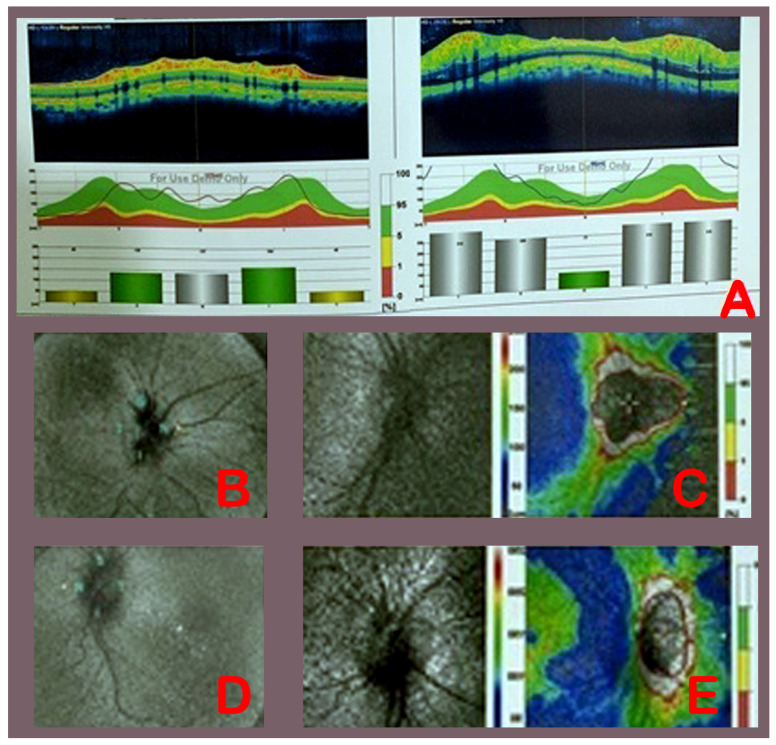
Pre-operative (**A**) OCT (RNFL, right eye 175 µm, left eye 212 µm). Pre-operative right (**B**) and left (**D**) eyes fundus and post-operative right (**C**) and left (**E**) eyes fundus and OCT (**B**) (RNFL, right eye 105 µm, left eye 107 µm) after ETV in patient no. 15.

In group B (ETV), pre-operative modified Frisen grading of papilledema right eyes were grade 1 in 1 patient, grade 2 in 2 patients, grade 3 in 5 patients, and grade 4 in 1 patient (Table 6). Post-operatively, 5 patients were grade 1 and 2 patients were grade 2 and 1 patient was grade 3 papilledema. In the same group B (ETV), in the left eyes, pre-operative modified Frisen gradings of papilledema were grade 1 in 1 patient, grade 2 in 3 patients, grade 3 in 2 patients, and grade 4 in 3 patients. Post-operatively, 5 patients were grade 1, 3 patients were grade 2, and 1 patient was grade 3. These changes of papilledema severity were not statistically significant in either the right (*p* value = 0.13) or left eyes (*p* value = 1.0). Figure 2 shows pre-operative eyes and post-operative eyes fundus and OCT in patient no. 15, who was treated with ETV.

Mean RNFL thickness differences (for both eyes) between pre-operative and post-operative periods (7 days after shunt surgeries) in Group A (VPS) and Group B (ETV) ere 48.1 ± 41.3 µm and 54.6 ± 28.1 µm, respectively. These differences between pre-operative and post-operative periods in both groups were not statistically significant (*p* value 0.56). Similarly, mean Frisen grading differences (for both eyes) between pre-operative and post-operative periods in Group A (VPS) (1.53 ± 0.87) and in Group B (ETV) (1.35 ± 1.11) were not statistically significant (*p* value 0.56).

## 4. Discussion

All 18 patients (100%) had papilledema and blurring of vision in both eyes at the time of enrolment into the study. The classical triad of raised ICP: headache, vomiting and visual disturbance were ubiquitous to any mode of presentation whether it was acute or chronic. In the present study, there were 4 (22.3%) cases of CPA tumor, 4 (22.3%) cases of posterior fossa tumor, 6 (33.33%) cases of aqueductal stenosis, and 4 (22.3%) cases of pineal tumor.

Chahlavi et al. [23] presented similar data regarding the etiology of adult-onset obstructive hydrocephalus. That study, regarding etiology of obstructive hydrocephalus, showed that space occupying lesions accounted for 58.9% and aqueductal stenosis accounted for 34.3% of obstructive hydrocephalus [23]. In a pre-operative fundoscopy of group A (VPS), Frisen grade 3 papilledema was commonly followed by grade 1 papilledema. After VPS, grade 1 papilledema was most common for both eyes which denotes a decrease in the severity of the papilledema after CSF diversion. Change of grading happened in 6 patients out of 9 in both eyes (66.67%) with a *p* value of 0.028 for right eye and 0.333 for left eye. On the other hand, pre-operative fundoscopy of group B (ETV) showed Frisen grade 3 and grade 4 papilledema were commonly followed by grade 2 papilledema. After ETV, grade 1 papilledema was common for both eyes, which denotes a decrease in the severity of papilledema after CSF diversion. Change of grading occurred in 5 patients out of 9 in both eyes (55.5%) with a *p* value of 0.13 for the right eye and 1.0 for the left eye. Rizzo et al. [24] showed CSF shunting resulted in decrement of Frisen papilledema grade by 2.19 ± 0.71 (*p* < 0.0001). As CSF diversion is supposed to relieve papilledema in obstructive hydrocephalus but may be associated with a transient or permanent absorptive component, it may hinder the complete resolution of raised intracranial pressure.

The absorptive component in the background of obstructive hydrocephalus influenced the grading change in papilledema [24]. The mean pre-operative RNFL thickness in group A for right eyes was 159.8 ± 59.4 µm, while the mean post-operative (7 days after shunt surgeries) RNFL thickness for right eyes was 128.4 ± 36.3 µm. The change in mean RNFL thickness carried a statistically significant level (*p*-value) of 0.016. In case of left eyes, the mean pre-operative RNFL thickness was 189.0 ± 56.6 µm and the mean post-operative RNFL thickness was 124.2 ± 27.6 µm. The change in mean RNFL thickness carried a statistically significant level (*p*-value) of 0.003. In contrast, the mean pre-operative RNFL thickness in group B for right eyes was 158.4 ± 30.7 µm, while the mean post-operative RNFL thickness for right eyes was 110.5 ± 27.5 µm. The change in mean RNFL thickness carried a statistically significant value (*p*-value) < 0.001. In the case of left eyes, the mean pre-operative RNFL thickness was 161.1 ± 45.2 µm and the mean post-operative RNFL thickness was 99.6 ± 21.6 µm. The change in mean RNFL thickness carried a statistically significant level (*p*-value) of 0.001. Koktekir et al. [25] reported a pre-operative RNFL thickness of 244.5 ± 53.4 microns and a post-operative RNFL thickness of 147.5 ± 54.1 microns following VP shunt surgery. They also showed a pre-operative RNFL thickness of 259.7 ± 35.8 microns and a post-operative RNFL thickness of 158.3 ± 21.1 microns in the case of ETV [25]. As the resolution of the papilledema is a good clinical indicator of decreased ICP after surgery, the existence of papilledema in the follow-up period is more predictive than radiological findings regarding failure of the surgical procedure [25,26,27,28,29]. In 2017, Alfonso et al. [30] proposed the use of OCT as a new diagnostic marker for idiopathic normal pressure hydrocephalus, reporting, however, that non-shunted idiopathic normal pressure hydrocephalus patients showed choroidal thinning in combination with normal RNFL values.

Rizzo et al. [24] showed that CSF shunting resulted in significant improvement in average retinal nerve fiber layer (RNFL) thickness measurements by optical coherence tomography, which decreased by 87.27 ± 16.65 microns (*p* < 0.0001), and Frisen papilledema grades, which decreased by 2.19 ± 0.71 (*p* < 0.0001). From the present data analysis, it was determiend that both surgical procedures (VPS and ETV) were able to decrease the severity of papilledema post-operatively. From this study, it appears that there is no superiority of choice to any surgical procedure in these patients with obstructive hydrocephalus in the early post-operative period. Similarly, Swanson et al. [31] determined that intracranial pressure correlated with maximal retinal nerve fiber layer thickness (*p* ≤ 0.001), maximal retinal thickness (*p* ≤ 0.001), and maximal anterior retinal projection (*p* = 0.003), as the length of vertical line from the maximal internal limiting membrane elevation to the margins of the peripapillary Bruch’s membrane. An attractive attribute of OCT as a surrogate measure of ICP is its repeatability over time [32,33,34]. OCT-derived measures of optic nerve head volume may be more sensitive to papilledema improvement than the Frisen scale for the purposes of monitoring the treatment of ICP [35]. True papilledema secondary to increased ICP is expected to fluctuate with ICP. The longitudinal behavior and variability of the OCT parameters and specific metrics may be less prone to anatomic variations; it holds promise as a potential discriminator between true papilledema and pseudopapilledema (defined as anomalous elevation of one or both optic discs without edema of the retinal nerve fiber layer) [36] and warrants future study [31,32].

Our study has several limitations. First of all, the present study presented a very small number of patients and represents a single-institution experience. Second, the heterogeneity of the patient population and the short follow-up (7 days), due to the fact that most of these patients requires tumor removal surgery as soon as possible, inevitably added another layer of selection bias. Multicenter and randomized studies are needed to better understand the CSF dynamics of these patients. The use of virtual reality and new technologies in neurosurgery, which is spreading more and more in neurosurgery [37,38,39,40,41], will probably help surgeons to better understand these CSF dynamics and the role of obstructive hydrocephalus in the development of papilledema.

## 5. Conclusions

The resolution of papilledema was analyzed both pre- and post-operatively by decrease in modified Frisen grading in fundoscopy and retinal nerve fiber layer thickness (RNFL), followed by non-invasive optical coherence tomography (OCT). According to this study, VPS and ETV were equally efficient in ameliorating papilledema in obstructive hydrocephalus. For this reason, both can be considered for this particular purpose, according to their own indications and contraindications, complications and single-center experience.

## Figures and Tables

**Table 1:** Modified Frisen grading of papilledema.

Grade 1	Grayish C-shaped halo surrounding the disc; sparing of the temporal disc margin; radial nerve fiber striation disruption.
Grade 2	Halo becomes circumferential; nasal border elevation; no major vessel obscuration.
Grade 3	Obscuration of at least one vessel leaving the disc; elevation of all borders; circumferential halo.
Grade 4	Obscuration of a major vessel on the disc; complete elevation including the cup; circumferential halo.
Grade 5	Obscuration of all vessels on the disc and leaving the disc; all features of Grade 4.

**Table 2:** Demographic data of study population.

Categories	Sub-Categories	Number (%)	
Age	Years	Group A (VPS)	Group B (ETV)
	18–24	3 (33.3%)	3 (33.3%)
	25–35	3 (33.3%)	3 (33.3%)
	36–50	3 (33.3%)	3 (33.3%)
Sex	Male	6 (66.7%)	7 (77.8%)
	Female	3 (33.3%)	2 (22.2%)
Etiology of obstructive hydrocephalus	CPA tumor	2 (22.2%)	2 (22.2%)
	Posterior fossa tumor	3 (33.3%)	1 (11.1%)
	Aqueductal stenosis	2 (22.2%)	4 (44.4%)
	Pineal tumor	2 (22.2%)	2 (22.2%)
Clinical presentation	Papilledema	9 (100%)	9 (100%)
	Headache	9 (100%)	9 (100%)
	Vomiting	6 (66.7%)	5 (27.7%)
	Gait disturbance	4 (44.4%)	2 (22.2%)
	Hearing loss	4 (44.4%)	2 (22.2%)

CPA, cerebellopontine angle.

**Table 3:** Modified Frisen grading and RNFL thickness in both eyes before and after VPS (Group A) or ETV (Group B).

Case No.	Surgical Treatment	Preop Frisen Rt	Preop RNFL Rt (µm)	Postop Frisen Rt	Postop RNFL Rt (µm)	Preop Frisen Lt	Preop RNFL Lt (µm)	Postop Frisen Lt	Postop RNFL Lt (µm)	RNFL Change Rt (µm)	RNFL Change Lt (µm)	Frisen Change Rt	Frisen Change Lt
1.	VPS	Grade 5	300	Grade 3	197	Grade 5	300	Grade 2	176	103	124	2	3
2.	VPS	Grade 3	151	Grade 1	105	Grade 1	124	Grade 1	107	46	17	2	0
3.	VPS	Grade 1	121	Grade 1	109	Grade 4	239	Grade 1	97	12	142	0	3
4.	VPS	Grade 3	180	Grade 2	171	Grade 3	199	Grade 3	165	9	34	1	0
5.	VPS	Grade 1	99	Grade 1	96	Grade 3	166	Grade 1	116	3	50	0	2
6.	VPS	Grade 1	116	Grade 1	102	Grade 3	176	Grade 1	106	14	70	0	2
7.	VPS	Grade 3	175	Grade 2	154	Grade 4	212	Grade 1	125	21	87	1	3
8.	VPS	Grade 3	165	Grade 1	119	Grade 2	165	Grade 1	119	46	46	2	1
9.	VPS	Grade 2	132	Grade 1	103	Grade 1	120	Grade 1	107	29	13	1	0
10.	ETV	Grade 2	107	Grade 1	90	Grade 2	103	Grade 1	64	17	39	1	1
11.	ETV	Grade 4	184	Grade 2	136	Grade 3	165	Grade 2	130	48	35	2	1
12.	ETV	Grade 3	175	Grade 2	144	Grade 4	212	Grade 1	115	31	97	1	3
13.	ETV	Grade 3	176	Grade 1	102	Grade 2	116	Grade 1	92	74	24	2	1
14.	ETV	Grade 3	175	Grade 1	104	Grade 4	215	Grade 1	102	71	113	1	3
15.	ETV	Grade 3	175	Grade 1	105	Grade 4	212	Grade 1	107	70	105	2	3
16.	ETV	Grade 2	149	Grade 1	103	Grade 2	143	Grade 1	107	46	36	1	1
17.	ETV	Grade 3	178	Grade 2	148	Grade 3	172	Grade 1	112	30	60	1	2
18.	ETV	Grade 1	107	Grade 1	63	Grade 1	112	Grade 1	68	44	44	0	0

ETV, endoscopic third ventriculostomy; RNLF, retinal nerve fiber layer; VPS, ventriculoperitoneal shunt.

**Table 4:** Change to RNFL thickness in Group A (VPS) and Group B (ETV).

Surgical Treatment	Eye	RNFL Thickness Mean ± SD (Micrometers)		*p* Value
		Pre-Operative	Post-Operative	
VPS	Right	159.8 ± 59.4	128.4 ± 36.3	0.016
VPS	Left	189.0 ± 56.6	124.2 ± 27.6	0.003
ETV	Right	158.4 ± 30.7	110.5 ± 27.5	<0.001
ETV	Left	161.1 ± 45.2	99.6 ± 21.6	0.001

ETV, endoscopic third ventriculostomy; RNLF, retinal nerve fiber layer; VPS, ventriculoperitoneal shunt.

**Table 5:** Change to Frisen grading 7 days after VPS (Group A).

Eye	Pre-Operative Frisen Grading	Post-Operative Frisen Grading			Total	Spearman Rho	*p* Value
		Grade 1	Grade 2	Grade 3			
Right	Grade 1	3	0	0	3	0.721	0.028
	Grade 2	1	0	0	1		
	Grade 3	2	2	0	4		
	Grade 5	0	0	1	1		
Left	Grade 1	2	0	0	2		
	Grade 2	1	0	0	1		
	Grade 3	2	0	1	3	0.375	0.333
	Grade 4	1	1	0	2		
	Grade 5	0	0	1	1		

**Table 6:** Change of Frisen grading 7 days after ETV (Group B).

Eye	Pre-Operative Frisen Grading	Post-Operative Frisen Grading			Total	Spearman Rho	*p* Value
		Grade 1	Grade 2	Grade 3			
Right	Grade 1	1	0	0	1	0.6	0.13
	Grade 2	2	0	0	2		
	Grade 3	2	2	1	5		
	Grade 4	0	0	1	1		
Left	Grade 1	1	0	0	1		
	Grade 2	2	1	0	3	0.7	1.0
	Grade 3	1	1	0	2		
	Grade 4	1	1	1	3		

## Data Availability

Not applicable.
